# Seed Germination and Seedling Growth Influenced by Genetic Features and Drought Tolerance in a Critically Endangered Maple

**DOI:** 10.3390/plants12173140

**Published:** 2023-08-31

**Authors:** Detuan Liu, Jiajun Yang, Lidan Tao, Yongpeng Ma, Weibang Sun

**Affiliations:** 1 Yunnan Key Laboratory for Integrative Conservation of Plant Species with Extremely Small Populations/Key Laboratory for Plant Diversity and Biogeography of East Asia, Kunming Institute of Botany, Chinese Academy of Sciences, Kunming 650201, China; liudetuan@mail.kib.ac.cn (D.L.); yangjiajun@mail.kib.ac.cn (J.Y.); taolidan@mail.kib.ac.cn (L.T.); 2 University of the Chinese Academy of Sciences, Beijing 100049, China

**Keywords:** *Acer yangbiense*, seedling performance, trade-off, conservation

## Abstract

Understanding the adaptation of plant species will help us develop effective breeding programs, guide the collection of germplasm, and improve the success of population restoration projects for threatened species. Genetic features correlate with species adaptation. *Acer yangbiense* is a critically endangered plant species with extremely small populations (PSESP). However, no information was available on its seed germination and seedling growth in populations with different genetic characteristics. In this study, we investigated seed germination and compared the performance of 566 seedlings in 10 maternal half-sib families cultivated in Kunming Botanical Garden. The results showed that *A. yangbiense* seeds required an average of 44 days to start germinating, with a 50% germination rate estimated to take about 47–76 days, indicating slow and irregular germination. There is a trade-off between the growth and survival in *A. yangbiense* seedlings, with fast growth coming at the cost of low survival. Groups that were able to recover from a recent bottleneck consistently had higher relative growth rates. High genetic diversity and low levels of inbreeding are likely to be responsible for their improved survival during drought conditions and rapid growth under optimal environmental conditions. Our results suggest that maternal genetic traits might be used as indicators for conservation and population restoration. These findings provide us with new information that could be applied to support ex situ conservation and reintroduction of threatened species.

## 1. Introduction

Seeds or seedlings are important germplasm resources and are often used in conservation programs for endangered species and in ecological projects to restore forest vegetation [1]. They are also commonly collected for botanical gardens and used in ex situ conservation, mainly because they are easy to obtain and propagate, and are more convenient to transport than larger adult individuals, particularly the remnants of tree species that have survived for hundreds of thousands of years. Of all the stages in the life cycle of trees, the seedling stage is the most vulnerable and sensitive to environmental stress [2], and seedlings have high drought-related mortality, particularly during germination and establishment [2]. Seedling survival is likely to be of critical importance to the regeneration of community dynamics and the persistence of population size in threatened tree species, and therefore, will affect both the distribution and persistence of the plant species [3,4], as well as the ability of the plant species to respond to stressful environments.

By the late 21st century, the global land area affected by drought is predicted to more than double [5]. Increased drought stress from climate change can affect species distributions, result in changes in biodiversity and community composition, reduce plant productivity, and cause widespread tree mortality [6,7]. Indeed, the widespread tree mortality that has occurred to date as a result of severe drought is not restricted to arid regions and is considered to be one of the principal threats to the function of terrestrial ecosystems [8,9].

Sessile plants have evolved different mechanisms to cope with the threat from drought environments [10]. High genetic diversity is usually positively related to the ability of species to adapt to stressful environments. In an endemic *Parashorea tomentella*, seedling survival increased with genetic diversity [11]. While inbreeding depression may increase the probability that recessive deleterious variants are exposed [12], promote the expression of homozygous deleterious alleles, and confer reduced fitness among the offspring of genetic relatives. Therefore, low levels of genetic diversity, high rates of harmful mutations, and high levels of inbreeding are particularly relevant for small or declining populations and could reduce the effective population size, limit a species’ ability to adapt, and ultimately cause eco-evolutionary extinction [13]. For example, lower genetic diversity and higher inbreeding have been associated with lower germination rates, shorter height, and a reduced early-life fitness of seedlings [14]. Population bottlenecks are closely correlated with a rise in stochasticity in small populations, which typically result in genetic diversity losses due to genetic drift [15]. Plant species with lower genetic diversity or lower rates of heterozygosity, higher numbers of homozygous deleterious mutations, and higher levels of inbreeding may therefore have lower fitness than those without [16]. While the effect of inbreeding on survival or short-term physiological responses of plants could not always be observed [17], in *Parashorea tomentella*, genetic diversity affected seedling survival but not growth or seed germination [11]. Seed germination, seedling survival, and growth are complexly affected by a variety of factors.

Despite extensive research on plant adaptation, little is known about how past population demographics, such as severe population bottlenecks, may potentially influence the ability of plants to adapt to current environmental stresses. This knowledge gap is mainly due to a lack of genetic data. In this study, we chose *A. yangbiense*, a maple species whose genetic features were investigated using resequencing data from a previous study [18], to address the following questions: (1) What are the characteristics of seed germination in different half-sib families with different genetic features? (2) Is it possible to use the genetic features of the maternal plants to predict the performance of the offspring? (3) Do plants with greater genetic diversity have a higher survival rate and relative growth rate? We hypothesized that the performance of *A. yangbiense* seedlings could be predicted by the genetic features of the maternal plants.

## 2. Results

### 2.1. Seed Traits and Germination

**Figure 1:**
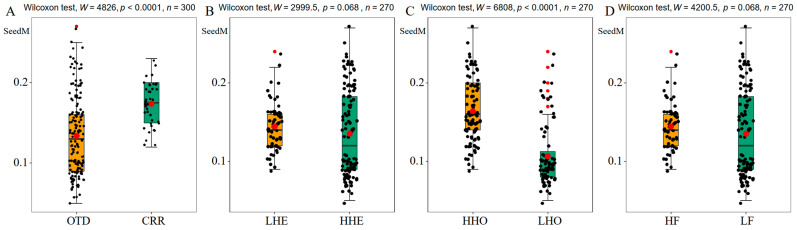
Seed mass difference among genetic groups in the 10 maternal families of *A. yangbiense*. (**A**), seed mass comparison between OTD and CRR groups; (**B**), seed mass comparison between LHE and HHE groups; (**C**), seed mass comparison between HHO and LHO groups; (**D**), seed mass comparison between HF and LF groups. Points in each panel represent individual seed mass (SeedM) and were jittered. Red dot on the bar represents the mean value and error bar represents the standard error.

Seed traits, including seed mass, seed width, seed length, wing width, and wing length, were variable among maternal families (half-sib families) (Table 1). The average seed mass was 0.14 g, ranging from 0.08 g in DYD2 and DYD3 to 0.21 g in BDH3. Seeds from the DYD population had the smallest seed mass, seed width, and seed length. The mean values of seed width, seed length, wing width, and wing length were 8.49 mm, 9.62 mm, 13.20 mm, and 32.85 mm, respectively, with mean cv values of 5.64%, 7.20%, 7.61%, and 7.65%. The mean cv value of seed mass was 13.66%, ranging from 7% in DYS1 to 18.21% in MLT1, demonstrating the high trait variation [19] of seed mass in *A. yangbiense*. Additionally, seed mass was significantly higher in the CRR and HHO groups, and high in the LHE and HF groups (Figure 1).

**Figure 2:**
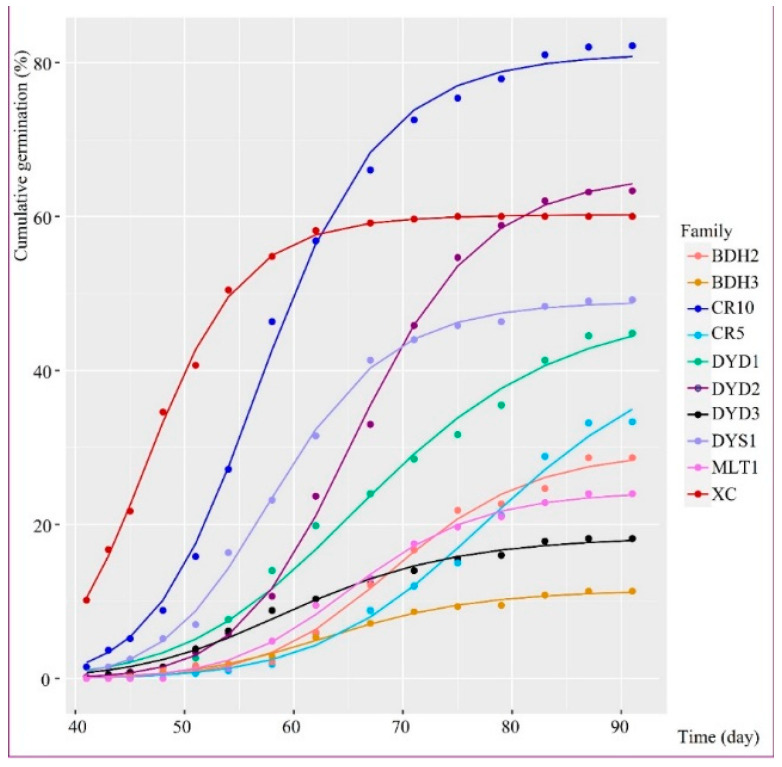
Cumulative germination in the 10 maternal families of *A. yangbiense*. BDH, Badahe population; CR, Chongren population; DYD, Diaoyudao population; DYS, Dayingshan population; MLT, Malutang population; XC, Xincun population.

*A. yangbiense* required an average of 44 days to begin germination (Table 2). The longest average germination time was found in maternal family MLT1, which took about 51 days to start germination. The shortest average germination time was found in the CR10, DYD2, and XC maternal families, which all took about 41 days to start germination. Mean T10, T50, and T90 for germination of *A. yangbiense* were 49.79 ± 0.01, 62.60 ± 2.99, and 77.07 ± 2.37, separately. Seed germination in *A. yangbiense* was slow and irregular (Figure 2). According to the results of the Auto-Germ tool, *A. yangbiense* took about 47–76 days to reach 50% germination, and around 72–84 days to reach 90% germination. *A. yangbiense* had a mean germination rate (GR) of 41%, ranging from 11% in BDH3 to 82% in CR10 (Table 2). The highest GR, GP (germination potential), and GI (germination index) were all found in the seedlings of CR10, and the lowest GR, GP, and GI were all found in seedlings of BDH3. The mean values of GR, GP, and GI were 0.41, 0.23, and 4.06, respectively. The mean cv value of germination was 15.19 ± 0.58.

### 2.2. Seedling Survival Rate

**Figure 3:**
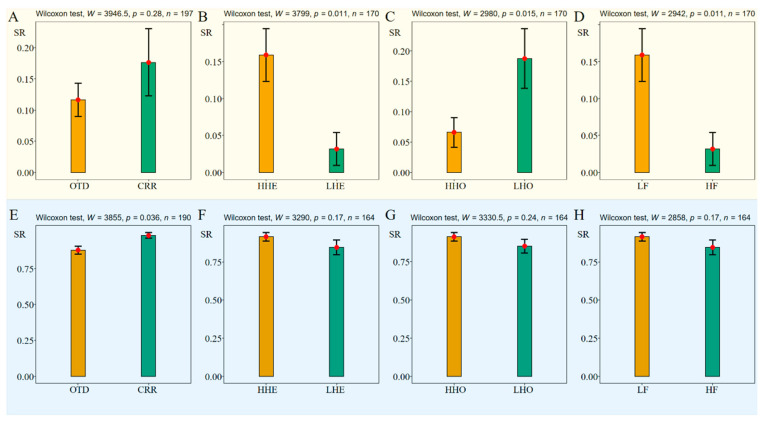
Comparison of survival status among genetic groups in the 10 maternal families of *A. yangbiense* under drought conditions (**A**–**D**) and under water conditions (**E**–**H**). The y axis represents SR (survival rate). Red dot represents the mean value and error bar represents the standard error.

*A. yangbiense* seedlings subjected to drought had a significantly higher death rate (~87%, 171/197) than those given water (11%, 22/194) (Figure S1, *W* = 4243, *p* < 0.001, *n* = 387). We compared the final survival rate (SR) among different genetic groups for the water and drought treatments, separately. In the drought treatment experiments (Figure 3A–D), the CRR group had a higher SR than did the OTD group (Figure 3A, *W* = 3946.5, *p* = 0.28, *n* = 197). As expected, SR was significantly higher in maternal families with high heterozygosity rates (the HHE group, Figure 3B, *W* = 3799, *p* = 0.011, *n* = 170), low numbers of homozygous deleterious mutations (the LHO group, Figure 3C, *W* = 2980, *p* = 0.015, *n* = 170), and low levels of inbreeding (the LF group, Figure 3D, *W* = 2942, *p* = 0.011, *n* = 170). In the water treatment experiments (Figure 3E–H), SR was significantly higher in the maternal families of the CRR group (Figure 3E, *W* = 3855, *p* = 0.036, *n* = 190), and high in maternal families of the HHE group (Figure 3F, *W* = 3290, *p* = 0.17, *n* = 164), the HHO group (Figure 3G, *W* = 3330.5, *p* = 0.24, *n* = 164), and the LF group (Figure 3H, *W* = 2858, *p* = 0.17, *n* = 164).

**Figure 4:**
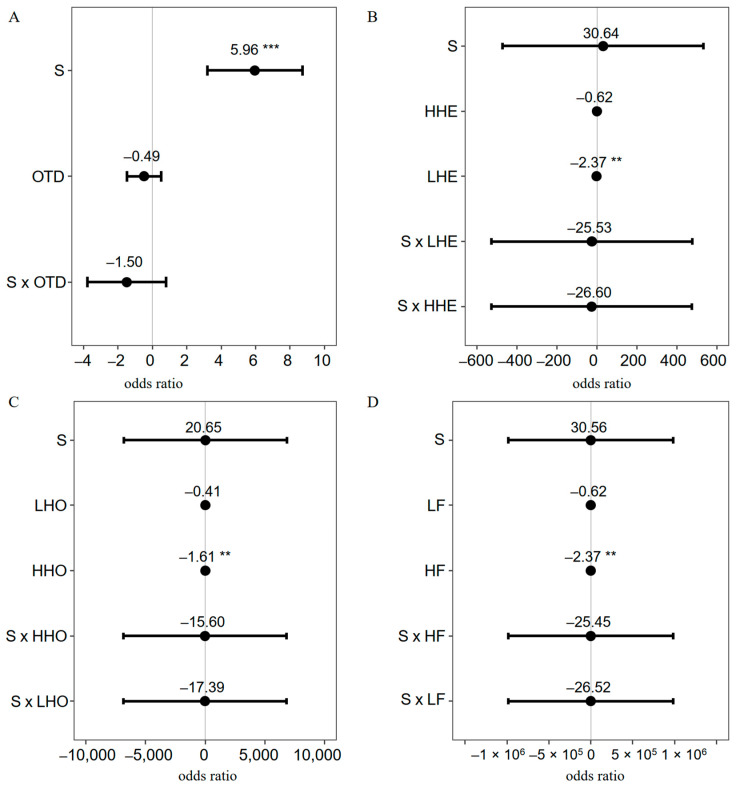
GLMM analysis of seedling survival status affected by treatment and genetic groups. (**A**), comparison of seedling survival between OTD and CRR groups; (**B**), comparison of seedling survival between LHE and HHE groups; (**C**), comparison of seedling survival between HHO and LHO groups; (**D**), comparison of seedling survival between HF and LF groups. “S” (water treatment), “G” (drought treatment), and “CRR” are category variables and are used as references (estimate = 0). “**”, *p* < 0.01, “***”, *p* < 0.001. The x axis represents the odds ratio (95% confidence). The mean odds ratio is shown on the confidence line.

When H1 (the first height measurement) and maternal individual were included as random effects, the results of the generalized linear mixed model (GLMM) showed that S (water treatment) had a positive effect on SR (Figure 4A–D) compared to drought treatment. The OTD group showed a lower SR than did the CRR group, but not significantly (Figure 4A, estimate = −0.49, *p* > 0.05). The LHE group (Figure 4B, estimate = −2.37, *p* < 0.01), the HHO group (Figure 4C, estimate = −1.61, *p* < 0.01), and the HF group (Figure 4D, estimate = −2.37, *p* < 0.01) had significantly lower SR than did the HHE, LHO, and LF groups, respectively, in the water treatment group compared with the drought treatment.

### 2.3. Relative Growth Rate

**Figure 5:**
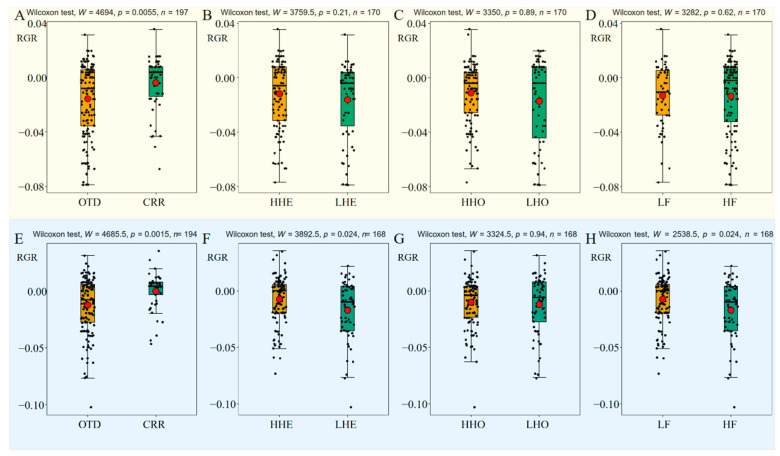
Comparison of relative growth rate among genetic groups in the 10 maternal families of *A. yangbiense* under drought (**A**–**D**) and water (**E**–**H**) treatments. Points in each panel represent individual relative growth rate (RGR) and were jittered. Red dot represents the mean value and error bar represents the standard error.

We compared the relative growth rate (RGR) among different genetic groups in the water and drought treatments, separately. Overall, the mean RGR in the water treatment group (about −0.93 mm/day) was greater than that in the drought treatment group (about −1.25 mm/day), but not significantly (Figure S2, *W* = 18374, *p* = 0.51, *n* = 391). RGR was consistently higher in the CRR group than in the OTD group (Figure 5A, *W* = 4694, *p* = 0.0005, *n* = 197; Figure 5E, *W* = 4685.5, *p* = 0.0015, *n* = 194), higher in the HHE group than in the LHE group (Figure 5B, *W* = 3759.5, *p* = 0.21, *n* =170; Figure 5F, *W* = 3892.5, *p* = 0.024, *n* = 168), higher in the HHO group than in the LHO group (Figure 5C, *W* = 3350, *p* = 0.89, *n* = 170; Figure 5G, *W* = 3324.5, *p* = 0.94, *n* = 168), and higher in the LF group than in the HF group (Figure 5D, *W* = 3282, *p* = 0.62, *n* = 170; Figure 5H, *W* = 2538.5, *p* = 0.024, *n* = 168), both in the drought and water treatment groups. We observed that the majority of seedlings suffered from dieback and a reduction in height as the upper stem perished from disease or weather damage [20].

**Figure 6:**
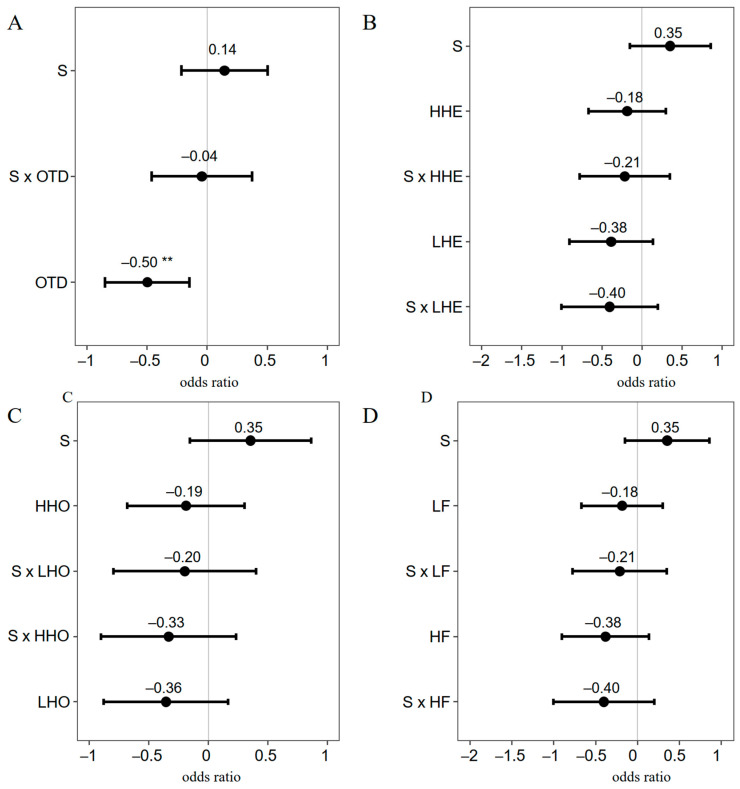
GLMM analysis of relative growth rate affected by treatment and genetic groups. (**A**), comparison of relative growth rate between OTD and CRR groups; (**B**), comparison of relative growth rate between LHE and HHE groups; (**C**), comparison of relative growth rate between HHO and LHO groups; (**D**), comparison of relative growth rate between HF and LF groups. “S” (water treatment), “G” (drought treatment), and “CRR” are category variables and are used as references (estimate = 0). “**”, *p* < 0.01. The x axis represents the odds ratio (95% confidence). The mean odds ratio is shown on the confidence line.

When H1 and maternal plants were included as random effects, water treatment had a positive effect on RGR (Figure 6A–D). Compared to the CRR group, the OTD group showed a significantly lower RGR (Figure 6A, estimate = −0.50, *p* < 0.01). The LHE group (Figure 6B, estimate = −0.38, *p* > 0.05), the LHO group (Figure 6C, estimate = −0.36, *p* > 0.05), and the HF group (Figure 6D, estimate = −0.38, *p* > 0.05) all showed lower RGR than did the HHE, HHO, and LF groups, respectively.

## 3. Discussion

### 3.1. Differences in Seed Traits and Seed Germination

Investigations into adaptation in endangered plant species, particularly at the vulnerable seed germination and subsequent seedling stages, will provide us with valuable information and inform future conservation decisions during global climate change [21]. To our knowledge, this study represents the first investigation of seedling performance in different intraspecific genetic groups, in particular at the level of population demographic history estimated with effective population size from tens of thousands of years ago based on resequencing data, as well as at the level of heterozygosity rate, deleterious mutations, and inbreeding.

Seed germination is one of the most important phenological traits affecting subsequent seedling growth [22]. The GRs of *A. yangbiense* were different among maternal families, for example, the GRs in maternal families, CR5 and CR10, were 33% and 82%, respectively. The average GR across the 10 maternal families of *A. yangbiense* was 41%, which is consistent with the findings of Yin et al. [23]. Previous research reported that *A. yangbiense* seeds are dormant and require four months of low temperature treatment to break dormancy [24]. It took an average of 47–76 days for *A. yangbiense* to reach 50% GR in our germination experiments, which is faster than *A. pycnanthum*, a vulnerable tree species from Japan, which took 16 weeks of cold stratification to reach a GR of 49–90% [25], and slower than *A. mono*, which took only about 21–28 days to reach 67–87% GR [26], although the experimental conditions were not identical. The slow, irregular germination of *A. yangbiense* may represent an adaptive strategy to cope with the monsoon climate in East Asia, which is characterized by very low rainfall during a long dry season from approximately November to May of the following year [27]. Seed dormancy and germination delay in some seeds could reduce the risk of exposure to adverse climate conditions during seasonal changes [28] and could be considered a bet-hedging strategy.

### 3.2. Growth-Survival Trade-Off

Impacted by monsoon climatic variability, China is significantly affected by drought hazards, particularly meteorological drought [29]. Our results showed that nearly all the *A. yangbiense* seedlings died after two months of growth under natural conditions with no water during the dry season at Kunming Botanical Garden, suggesting that drought has a significant effect on seedling survival (SR) in this species, which may be a factor determining its threatened status. The implementation of protection or restoration practices requires an understanding of their drought tolerance and adaptation. We therefore propose that the best time to reintroduce *A. yangbiense* populations would be during the rainy season, as this could increase the success rate of the restoration efforts by increasing the likelihood of seedling survival. This period corresponds to the growing season of this deciduous tree, and to the phase of the Chinese solar calendar known as “Grain in Ear”, which describes a period of markedly high temperatures, abundant rainfall, and high humidity beginning around 5 or 6 June each year. Follow the rhythm of nature and nature will reward you during the seedling reintroduction. 

**Figure 7:**
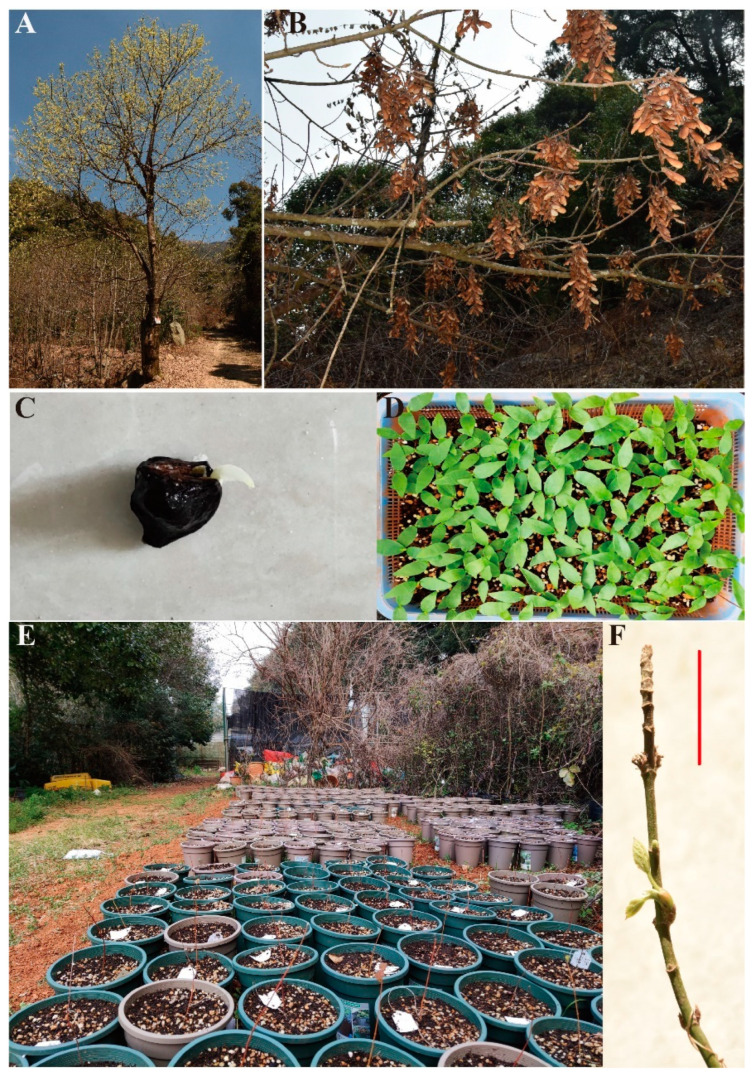
Overviews of *A. yangbiense*. (**A**) flowering individual; (**B**) mature seeds; (**C**) germinated seed; (**D**,**E**) seedlings; (**F**) dieback marked by red line.

In our experiments, almost all *A. yangbiense* seedlings experienced dieback (Figure 7F) in both the water and drought treatments, as indicated by their negative RGR. This may be another adaptive strategy by which *A. yangbiense* reacts to drought stress, including soil and atmospheric water deficits.

Genetic diversity is essential for the adaptability of a species, though there is no general relationship between genetic diversity and various fitness components, a lower level of heterozygosity may lead to a reduction in fitness [30]. For example, a positive correlation was found between population growth rate and allelic richness in an endangered orchid [31], favorable climate conditions may have facilitated the growth of juvenile pine individuals with higher genetic diversity [32], reduced heterozygosity is associated with reduced seedling survival in a tropical forest tree species [11], and lower genetic diversity increased the probability of mortality [33]. In our study, SR was significantly lower in the LHE, HHO, and HF groups in the drought treatments, indicating that *A. yangbiense* seedlings with high genetic diversity (HHE group), low number of homozygous deleterious mutations (LHO group), and low level of inbreeding (LF group) are better able to withstand a drought environment than those with low genetic diversity (LHE group), high number of deleterious mutations (HHO group), and high level of inbreeding (HF group). However, maternal families with significantly higher SR in the drought treatments did not always have significantly higher SR in the water treatments, indicating that seedling performance cannot always be predicted by the genetic features, depending on the fitness indicator (e.g., SR, RGR) and experimental treatments (e.g., water or drought). 

Growth and survival are key characters of forest dynamics and are important for population restoration. It has been reported that for small trees, there is a trade-off between rapid growth under favorable conditions and low mortality under unfavorable conditions [34]. In our study, *A. yangbiense* may allocate more resources to survival than to growth under drought stress, representing a trade-off between growth and mortality. The CRR group, which was previously predicted to be able to recover from a severe demographic bottleneck [18], consistently had higher RGR in both the drought and water treatments. In addition, both the HHE and LF groups had higher RGR in the water treatment, but not in the drought treatment, indicating that *A. yangbiense* seedlings with high genetic diversity and low levels of inbreeding grow faster under suitable conditions, and that plant species with high genetic diversity are likely to be more adaptable to increasing environmental stress than those with low diversity.

### 3.3. The Limitations of Our Research

As individuals from the same maternal lineage are more similar in genetically regulated traits than those chosen at random, the seedlings from the same mother tree are considered half siblings, and the genetic features of the mother plants were used as an indicator of their offspring [35]. Because all seedlings of *A. yangbiense* had a known mother, but an unknown father, we used only the genetic features of the mother plants to represent the genetic features of all their offspring. However, the genetic diversity, number of homozygous deleterious mutations, and level of inbreeding in plant seedlings can be affected by selection, purging, drift, recombination, and gene flow, and are therefore likely to be variable and uncertain. Future research on the genetic attributes of these offspring should be used to thoroughly address the complexity of this issue.

The sample size in this study is small, with only 10 maternal plants sampled due to the fact that few females could be found in the remaining populations, which is commonly a challenge when working with a critically endangered plant species with extremely small populations. Consequently, the findings of this study are relatively limited. 

## 4. Materials and Methods

### 4.1. Study Species

*A. yangbiense* (Figure 7A), which belongs to *Acer* sect. *Lithocarpa* Pax [36], is endemic to Yunnan Province, China. *A. yangbiense* is a plant species with extremely small populations (PSESP) and is categorized as Endangered on the IUCN Red List [37] and Critically Endangered on the Red List of Higher Plants in China [38]. Thorough field investigations in Yunnan revealed 577 mature individuals of *A. yangbiense*, and the species has a very restricted distribution range in Yunnan [39]. The chromosome-level genome of *A. yangbiense* has been reported, and the genetic parameters, including demographic history, deleterious mutations, and inbreeding coefficient, have been thoroughly investigated in 105 individuals of this species [18]. The seedling stage was considered to be the most crucial stage in limiting population regeneration [40]. Most *Acer* species, including *A. yangbiense*, prefer humid environments based on field observation. Species distribution modeling also suggested that annual precipitation contributed most to the suitability of potentially suitable habitat for most maple species [41].

Because *A. yangbiense* is andromonoecious, with an extremely low female to male ratio, most individuals are male and unable to bear fruit leading to a low fruit set rate [39]. To investigate the adaptation of *A. yangbiense* seedlings to drought stress, we collected mature seeds (Figure 7B) from as many ten maternal individuals from six populations (Table S1) in mid-November 2020. We used the maternal genetic traits as a proxy for the genetic traits of their half-sib families (offspring). We performed seed germination experiments, and further compared seedling growth and survival status with experiments at Kunming Botanical Garden, Chinese Academy of Sciences. In this study, the water factor was selected and considered separately, as combinations of impacts may either exacerbate or mitigate the observed effects [42].

### 4.2. Seed Traits and Germination Experiments

After collection, *A. yangbiense* fruits were air dried at room temperature in the laboratory until the germination experiments started. Because there was obvious morphological variation between *A. yangbiense* seeds from different maternal families, we randomly selected 30 seeds from each maternal family and measured seed length, seed width, wing width, and wing length following methods described previously [40]. We also weighted 20 intact seeds from each maternal family. The mean values and cv (coefficients of variation) were also calculated for these traits.

We randomly selected 600 seeds from each maternal individual (a total of 6000 seeds) for germination experiments on 19 January 2021, following a previous method [23]. Briefly, seeds were mixed with tap water and perlite, sealed in a plastic bag, and stratified in the top layer of a refrigerator (10 °C) to break the seed dormancy. The bags were taken out once a week and placed in the laboratory at ~20 °C for about eight hours, then put back and stratified again. When the radicle extended at least 5 mm, the seed was considered germinated and was taken out of the plastic bag (Figure 7C). Germinated seeds were counted and were then grown in a nursery at Kunming Botanical Garden (Figure 7D). The germination experiments lasted for about 90 days and were ceased on 20 April 2021 after no new germinated seeds had been observed for one week.

Germination onset time (the time required to start germination from the beginning of stratification to the first germination, T0), germination rate (the number of germinated seeds as a percentage of the total number of seeds, GR), germination index (the sum of the proportion of seeds germinated on the first day to the corresponding germination day, GI), and germination potential (the number of seeds germinated on their maximum germination day divided by the total number of experimental seeds, GP) were calculated following previous research [43,44]. We also calculated values for T10 (time required to reach 10% germination of total seeds), T50 (time required to reach 50% germination of total seeds), and T90 (time required to reach 90% germination of total seeds), as well as cv values using the Auto-Germ tool [45].

### 4.3. Common Garden Experiments

To eliminate the effect of soil heterogeneity, all seedlings were grown in the nursery with similar substrate (peat:coconut hust:perlite:kanuma = 3:1:1:1) and watered frequently using tap water. Fungicide was applied once to eliminate fungal diseases due to its susceptibility to fungal infection [18], and weeds were regularly removed to reduce vegetative competition. On 15 August 2021, seedlings were transplanted into plastic pots and moved from the nursery to an outdoor area near the nursery. A total of 571 seedlings (32–63 offspring for each maternal individual) were planted and tagged (Figure 7E). As far as was possible, we selected seedlings of a similar size for planting experiments. Transplanting of the seedlings was finished in a single day. All plants were fully watered after transplanting and were routinely watered with tap water about twice a week, depending on the soil moisture. Ten days after transplanting, dead seedlings were removed, leaving a total of 566 living seedlings. This mortality was assumed to be due to transplant shock [46]. Seedlings whose stems had a living part less than 2 cm in length were considered to be dead. We performed the first measurement of seedling height (H1) from soil surface to the tallest part of the main stem on 25 August 2021 (t1). The seedling pots were rearranged three times: once in December 2021, once on 15 March 2022, and once on 17 April 2022, to minimize the effects of pot position.

We next explored the effects of natural drought on the performance of *A. yangbiense* seedlings. After the last watering (16 March 2022), we randomly divided the surviving seedlings into two groups, one group was watered frequently (about twice a week) using tap water (the water experiment group) to keep the soil moist, and the other group was not watered until the end of the experiment (the natural group). South China is subject to a significant monsoon climate characterized by an arid season from November until the end of May, and the natural group was considered to be under drought stress. The final survival status (SR) of individuals at the end of the experiment (10 June 2022) was recorded as follows: survival to the end of the experiment = 1, dead at the end of the experiment = 0 [47]. The last measurement of seedling height (H2) was made on 6 May 2022 (t2). We defined the relative growth rate (RGR) as the difference in seedling height change in centimeters (cm) divided by the time interval in days (RGR = (H2 − H1)/(t2 − t1)). SR and RGR were used to represent seedling fitness of *A. yangbiense*.

### 4.4. Statistics

All statistical analyses were conducted in RStudio v 2022.2.3.492 [48] using R v 4.1.0 [49]. Results of seed length, width, and weight are presented as mean ± sd (standard deviation). Coefficient of variation (cv = sd/mean) was used to describe trait variations among maternal families [43]. Correlation coefficients were used to quantify associations among seed traits. We selected seed mass, which exhibited a strong correlation between seed trait pairs (Figure S3), for subsequent analysis.

FROH (frequency of runs of homozygosity), calculated by dividing the number of ROHs (runs of homozygosity) by the genome size, is often used as a coefficient of genomic inbreeding [50]. To investigate the effects of demographic history, heterozygosity rate, homozygous deleterious mutation, and FROH on adaptation in *A. yangbiense*, we divided the germinated seedlings into different half-sib groups according to the genetic features of their mother trees (Table 3) using the following criteria: (1) grouped according to the demographic history [18] into the CRR group (from the CR population in Yunlong County, which shows evident recovery after the most recent bottleneck, when a large population is dramatically reduced in size) and the OTD group (from other populations in Yangbi County, which shows a continuous decline in effective population size after the most recent bottleneck); (2) grouped by the mean value of the heterozygosity rate [18] into the high heterozygosity rate (HHE) group and the low heterozygosity rate (LHE) group; (3) grouped according to the mean value of the number of homozygous deleterious mutations [18] into the high homozygous deleterious mutation group (HHO) and the low homozygous deleterious mutation group (LHO); (4) grouped according to the mean value of FROH [18] into the high FROH group (HF) and the low FROH group (LF). Following previous methods [51], Wilcoxon’s nonparametric tests were performed using the wilcox_test function in rstatix v 0.7.0 [52] to compare the difference in seed mass, SR, and RGR among different pairs of groups.

We also used a generalized linear mixed model (GLMM) to test the effect of treatment (“G”: the drought experiment, and “S”: the water experiment), and the genetic characteristics and their interactions on the survival (SR) and relative growth rate (RGR) of the ten tested maternal families of *A. yangbiense*. Maternal individual (M) and the first height measurement (H1) were included as random effects within our models. We used the R software with the glmer function in the lme4 [53] to perform the GLMM. SR was binary and fitted using Binomial family, e.g., glmer (SR~Group × Group1 + (1|H1:M), family = binomial (link = “logit”)). RGR was assumed to follow a Gaussian distribution, e.g., glmer (RGR ~ Group × Group1 + (1|H1:M), family = gaussian (link = “identity”)). The 95% confidence was estimated and plotted with the plot_model function in the sjPlot v 2.8.9 [54] and was used to represent the effect of variables.

## 5. Conclusions

The seedling stage of *A. yangbiense* is vulnerable to dry weather in early summer. Under experimental conditions, *A. yangbiense* took an average of 44 days to begin germinating, and a 50% germination rate was reached after an estimated 47–76 days. This slow and irregular germination, along with seedling dieback, might represent a series of drought adaptation strategies that have evolved in *A. yangbiense* in response to dry seasons. *A. yangbiense* seedlings prioritize resource allocation for survival over their ability to grow under drought stress, demonstrating a trade-off between growth and survival. Maternal genetic features of *A. yangbiense* might be used as indicators for conservation efforts in the face of climate change. The lower mortality under drought stress and faster growth under optimal conditions of *A. yangbiense* seedlings may likely be explained by the mother plants with high genetic diversity and low inbreeding levels. The CRR group consistently exhibited higher RGR than the OTD group in both water and drought experiments. These findings could be used for future conservation and restoration of *A. yangbiense* populations.

## Figures and Tables

**Table 1:** Seed trait variance of the 10 maternal families of *A. yangbiense*.

M	SeedM	cv%	SeedW	cv%	SeedL	cv%	WingW	cv%	WingL	cv%
BDH2	0.17 ± 0.02 bc	13.95	9.55 ± 0.46 b	4.83	10.26 ± 0.53 bc	5.21	14.58 ± 1.04 b	7.12	40.17 ± 2.67 a	6.64
BDH3	0.21 ± 0.03 a	13.55	10.27 ± 0.49 a	4.81	10.77 ± 0.88 ab	8.17	16.23 ± 1.44 a	8.86	35.88 ± 2.96 b	8.26
CR5	0.18 ± 0.03 b	16.83	8.58 ± 0.66 c	7.72	9.32 ± 1.19 d	12.77	16.14 ± 1.07 a	6.61	32.95 ± 3.42 d	10.39
CR10	0.17 ± 0.03 b	15.28	9.54 ± 0.48 b	5.05	11.13 ± 0.84 a	7.53	15.98 ± 1.16 a	7.25	37.12 ± 2.63 d	7.08
DYD1	0.10 ± 0.01 de	8.91	7.86 ± 0.36 d	4.61	8.74 ± 0.47 d	5.42	10.30 ± 0.61 de	5.95	30.93 ± 1.87 d	6.03
DYD2	0.08 ± 0.01 e	11.04	6.71 ± 0.38 e	5.67	7.75 ± 0.45 e	5.80	10.81 ± 0.66 de	6.13	27.79 ± 1.69 e	6.08
DYD3	0.08 ± 0.01 e	17.45	6.55 ± 0.32 e	4.92	7.90 ± 0.34 e	4.30	9.97 ± 0.82 e	8.20	26.74 ± 2.18 e	8.14
DYS1	0.15 ± 0.01 c	7.00	8.04 ± 0.70 d	8.67	8.94 ± 0.87 d	9.74	10.84 ± 1.18 d	10.91	33.07 ± 3.11 cd	9.40
MLT1	0.17 ± 0.03 bc	18.21	9.62 ± 0.54 b	5.64	11.31 ± 0.62 a	5.47	14.73 ± 1.16 b	7.88	35.19 ± 2.58 bc	7.33
XC1	0.12 ± 0.02 d	14.37	8.16 ± 0.37 d	4.50	10.06 ± 0.76 c	7.54	12.46 ± 0.89 c	7.16	28.66 ± 2.04 e	7.12
Mean	0.14	13.66	8.49	5.64	9.62	7.20	13.20	7.61	32.85	7.65

M, maternal individual; BDH, Badahe population; CR, Chongren population; DYD, Diaoyudao population; DYS, Dayingshan population; MLT, Malutang population; XC, Xincun population; seedM, seed mass (g); seedW, seed width (mm); seedL, seed length (mm); wingW, wing width (mm); wingL, wing length (mm). Seed traits are expressed as mean value ± standard deviation; cv, coefficient of variation. The letters after the values show the significant differences tested by LSD (Least Significant Difference), and the *p*-value has been adjusted by the Bonferroni method.

**Table 2:** Statistics of seed germination in the 10 maternal families of *A. yangbiense*.

M	T0 (Day)	GR (%)	GP (%)	GI	Cv (%)	T10 (Day)	T50 (Day)	T90 (Day)
BDH2	45	29	12	2.46	13.88	58.7	68.9	84.1
BDH3	45	11	5	1.05	16.31	50.1	62.9	81.1
CR5	45	33	29	2.71	12.83	59.5	76.1	84.1
CR10	41	82	46	8.36	16.73	47.5	56.9	73.0
DYD1	43	45	14	4.06	17.10	52.1	65.1	82.3
DYD2	41	63	24	5.71	13.04	54.4	66.3	77.2
DYD3	43	18	9	1.78	18.45	48.4	58.7	79.8
DYS1	43	49	41	4.93	15.83	47.7	58.7	71.5
MLT1	51	24	10	2.15	13.54	55.2	65.6	80.3
XC	41	60	35	7.42	14.15	24.2	46.9	57.2
Mean	44	41	23	4.06	15.19 ± 0.58	49.79 ± 0.01	62.60 ± 2.99	77.07 ± 2.37

M, maternal population; BDH, Badahe population; CR, Chongren population; DYD, Diaoyudao population; DYS, Dayingshan population; MLT, Malutang population; XC, Xincun population; T0, germination onset time; GR, germination rate; GI, germination index; GP, germination potential; cv, coefficient of variation; T10, time to reach 10% germination; T50, median germination time, time to reach 50% germination; T90, time to reach 90% germination.

**Table 3:** Genetic parameters and groups of the 10 maternal individuals of *A. yangbiense*.

M	D	HetRate	NHom	FROH	Group1	Group2	Group3	Group4
BDH2	0	0.46	1164	0.15	OTD	HHE	HHO	LF
BDH3	0	0.51	1072	0.12	OTD	HHE	HHO	LF
CR5	1	0.40	1135	0.12	CRR	HHE	HHO	LF
CR10	1	/	/	/	CRR	/	/	/
DYD1	0	0.39	1005	0.21	OTD	HHE	LHO	LF
DYD2	0	0.45	961	0.12	OTD	HHE	LHO	LF
DYD3	0	0.39	970	0.20	OTD	HHE	LHO	LF
DYS1	0	0.27	1198	0.39	OTD	LHE	HHO	HF
MLT1	0	0.23	591	0.61	OTD	LHE	LHO	HF
XC1	0	0.34	1235	0.32	OTD	LHE	HHO	HF

M, maternal individual; BDH, Badahe population; CR, Chongren population; DYD, Diaoyudao population; DYS, Dayingshan population; MLT, Malutang population; XC, Xincun population. D, demographic history; 0 represents a population that showed a declining trend following the recent bottleneck event, and 1 represents a population that was able to recover after the recent bottleneck event. HetRate, heterozygosity rate; NHom, number of homozygous potentially deleterious mutations; FROH, frequency of runs of homozygosity, an indicator of inbreeding level; “/”, missing value. Demography, HetRate, NHom, and FROH were all obtained from Ma et al. [18]. OTD, a maternal family from a population that declined continuously after the recent bottleneck event; CRR, a maternal family from the CR population that showed recovery after the recent bottleneck event; HHE, a maternal family from maternal plants with a high rate of heterozygosity; LHE, a maternal family from maternal plants with a low rate of heterozygosity; HHO, a maternal family from maternal plants with high numbers of homozygous deleterious mutations; LHO, a maternal family from maternal plants with low numbers of homozygous deleterious mutations; HF, a maternal family from maternal plants with high levels of inbreeding (FROH); LF, a maternal family from maternal plants with low levels of inbreeding (FROH).

## Data Availability

The data presented in this study are available on request from the corresponding author.

## Supplementary Materials

The following supporting information can be downloaded at: https://www.mdpi.com/article/10.3390/plants12173140/s1, Figure S1: Comparison of survival rate between the drought and the water groups of *Acer yangbiense*; Figure S2: Comparison of relative growth rate between the drought and the water groups of *A. yangbiense*; Figure S3: Pearson’s correlation coefficients among seed trait and genetic feature pairs; Table S1: Distribution of the 10 maternal individuals of *A. yangbiense*.
