# Supplementary material for: Seed Germination and Seedling Growth Influenced by Genetic Features and Drought Tolerance in a Critically Endangered Maple
Source: Plants (Basel). 2023 Aug 31;12(17):3140. doi: 10.3390/plants12173140 (PMC10490246; doi:10.3390/plants12173140)
Supplement: Supplementary file 1 [file plants-12-03140-s001.zip › plants-2514886-supplementary.pdf]

Seed germination and seedling growth influenced by genetic features and drought tolerance in a critically endangered maple

Detuan Liu, Jiajun Yang, Lidan Tao, Yongpeng Ma and Weibang Sun

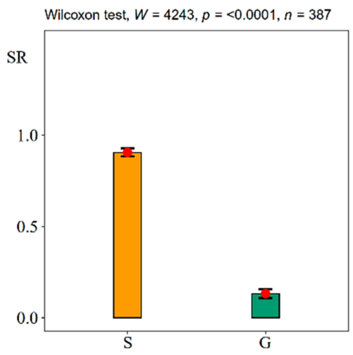

Figure S1. Comparison of survival rates between the drought and the water groups of *Acer yangbiense*. SR, survival rate; S, water group; D, drought group. Red dots represent the mean value, error bars represent the standard error.

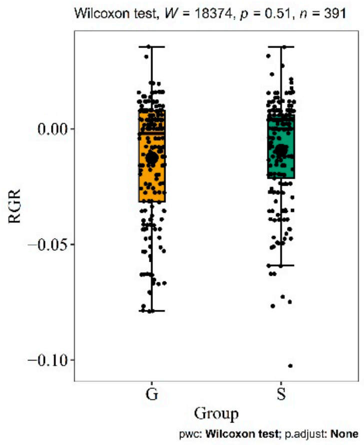

Figure S2. Comparison of relative growth rate between the drought and the water groups of *A. yangbiense*. RGR, relative growth rate; S, water group; G, drought group. Error bars represent the standard error.

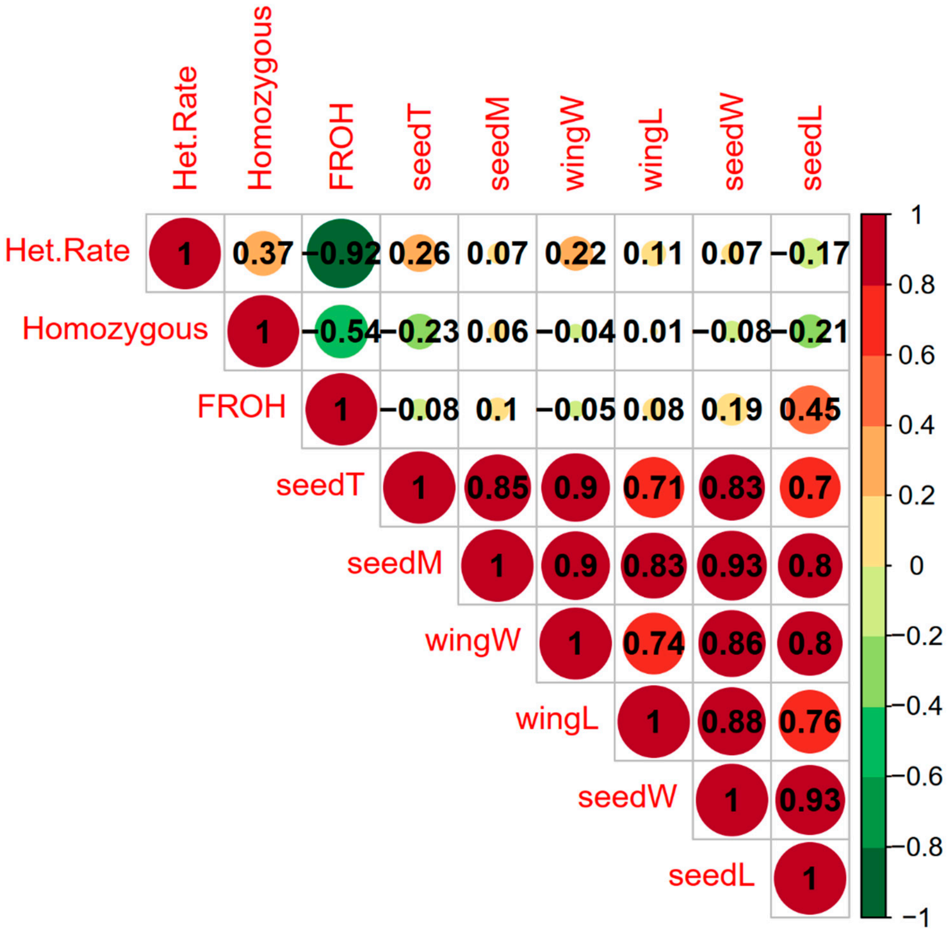

Figure S3. Pearson's correlation coefficients among seed trait and genetic feature pairs. seedM, seed mass (g); seedW, seed width (mm); seedL, seed length (mm); wingW, wing width (mm); wingL, wing length (mm); seedT, seed thickness (mm); het.Rate, heterozygosity rate; Homozygous, number of homozygous potentially deleterious mutations; The colour of the circles indicates the magnitude of the Pearson's correlation coefficients, with cold colour indicating a negative and warm colour indicating a positive relationship.

Table S1. Distribution of the 10 maternal individuals of *A. yangbiense*.

M	Latitude	Longitude	Altitude	Location
BDH2	25°36'27.76"	99°53'19.87"	2456	Badahe, Yangbi
BDH3	25°36'27.40"	99°53'18.49"	2461	Badahe, Yangbi
CR5	25°57'32.87"	99°02'10.23"	2283	Chongren, Yunlong
CR10	25°57′38.54″	99°02′41.92″	2458	Chongren, Yunlong
DYD1	25°35'8.16"	99°53'21.48"	2381	Diaoyudao, Yangbi
DYD2	25°35'8.16"	99°53'21.48"	2381	Diaoyudao, Yangbi
DYD3	25°35'8.16"	99°53'21.48"	2381	Diaoyudao, Yangbi
DYS1	25°34'16.50"	99°52'45.79"	2297	Dayingshan, Yangbi
MLT1	25°44'56.00"	100°00'23.00"	2427	Malutang, Yangbi
XC1	25°34'51.09"	99°55'25.26"	2164	Xincun, Yangbi

Note: M, maternal individual; BDH, Badahe population; CR, Chongren population; DYD, Diaoyudao population; DYS, Dayingshan population; MLT, Malutang population; XC, Xincun population.
